# Enteric Microbiome Metabolites Correlate with Response to Simvastatin Treatment

**DOI:** 10.1371/journal.pone.0025482

**Published:** 2011-10-13

**Authors:** Rima Kaddurah-Daouk, Rebecca A. Baillie, Hongjie Zhu, Zhao-Bang Zeng, Michelle M. Wiest, Uyen Thao Nguyen, Katie Wojnoonski, Steven M. Watkins, Miles Trupp, Ronald M. Krauss

**Affiliations:** 1 Duke University Medical Center, Durham, North Carolina, United States of America; 2 Rosa and Company, Cupertino, California, United States of America; 3 Department of Statistics and Bioinformatics Research Center, North Carolina State University, Raleigh, North Carolina, United States of America; 4 Department of Statistics, University of Idaho, Moscow, Idaho, United States of America; 5 Lipomics Technologies-Tethys Bioscience, West Sacramento, California, United States of America; 6 Bioinformatics Research Group, AI Center, SRI International, Menlo Park, California, United States of America; 7 Children's Hospital Oakland Research Institute, Oakland, California, United States of America; The University of Hong Kong, Hong Kong

## Abstract

Although statins are widely prescribed medications, there remains considerable variability in therapeutic response. Genetics can explain only part of this variability. Metabolomics is a global biochemical approach that provides powerful tools for mapping pathways implicated in disease and in response to treatment. Metabolomics captures net interactions between genome, microbiome and the environment. In this study, we used a targeted GC-MS metabolomics platform to measure a panel of metabolites within cholesterol synthesis, dietary sterol absorption, and bile acid formation to determine metabolite signatures that may predict variation in statin LDL-C lowering efficacy. Measurements were performed in two subsets of the total study population in the Cholesterol and Pharmacogenetics (CAP) study: Full Range of Response (FR), and Good and Poor Responders (GPR) were 100 individuals randomly selected from across the entire range of LDL-C responses in CAP. GPR were 48 individuals, 24 each from the top and bottom 10% of the LDL-C response distribution matched for body mass index, race, and gender. We identified three secondary, bacterial-derived bile acids that contribute to predicting the magnitude of statin-induced LDL-C lowering in good responders. Bile acids and statins share transporters in the liver and intestine; we observed that increased plasma concentration of simvastatin positively correlates with higher levels of several secondary bile acids. Genetic analysis of these subjects identified associations between levels of seven bile acids and a single nucleotide polymorphism (SNP), rs4149056, in the gene encoding the organic anion transporter SLCO1B1. These findings, along with recently published results that the gut microbiome plays an important role in cardiovascular disease, indicate that interactions between genome, gut microbiome and environmental influences should be considered in the study and management of cardiovascular disease. Metabolic profiles could provide valuable information about treatment outcomes and could contribute to a more personalized approach to therapy.

## Introduction

Statins are HMG-CoA reductase inhibitors that are widely used to reduce plasma levels of LDL cholesterol (LDL-C) and the risk for coronary artery disease (CAD) [1], [2], [3], [4]. However, statins also have a number of other important biological actions that may contribute to treatment benefit (e.g,, reducing inflammation) or adverse events, (e.g., myopathy, increased risk for type 2 diabetes mellitus [5]. Furthermore, efficacy of statins for lowering LDL-C and for reducing CAD risk can vary greatly among individuals [6]. For these reasons, identification of pre-treatment metabolic signatures or “biomarkers” predictive of response would be useful for targeting this drug to the population that may benefit most from its use.

Metabolomics brings powerful tools for mapping the metabolic state of individuals prior to treatment and global biochemical changes induced by drug treatment [7], [8], [9], [10]. Recently we reported that baseline metabolic profiles so called metabotypes do inform about trajectory of response to antidepressants escitalopram and sertraline [7], [8]. A new field is evolving called Pharmacometabolomics (also pharmacometabonomics) for applications of metabolomics in personalized medicine [10] and where biochemical data is being used to inform about treatment outcomes.

While it is well established that response to therapeutics can be affected by genetic and environmental factors, the enteric microbiome might also play a role in uncharacterized ways. Metabolomics provides a unique method to characterize the net interactions among these contributing factors. Numerous studies, by members of this group and others, have identified genetic polymorphisms that contribute to variability in the LDL-C response to statins [6], but only a small proportion of the variance has been explained by these factors [11]. Some statins are administered as inactive precursor drugs that are activated by endogenous biotransformation pathways, and there is increasing interest in the role of gut bacteria in the metabolism of drugs [12] including simvastatin. Metabolomics can capture a unique portrait of the state and changes of the gut microbiota by direct measurements of metabolite production [12], [13], [14]. Recently several metabolites produced by the gut microbiome were implicated in cardiovascular disease [15], highlighting for the first time an important and complementary role that the gut microbiome plays in cardiovascular health, and indicating the need to study net interactions between genome, gut microbiome and the environment.

Given that the primary cellular action of statins is well upstream of the factors that modulate plasma lipoprotein metabolism, there are many potential pathways that may act to influence the magnitude of the statin response. In this study, we sought to identify metabolites whose plasma levels predict LDL-C lowering response to statin treatment (simvastatin 40 mg/day) using a defined metabolomic approach. We measured a targeted panel of metabolites related to cholesterol metabolism in three known pathways: cholesterol synthesis, dietary sterol absorption, and bile acid formation. We detected significant correlations between gut-derived metabolites and simvastatin response. This supports further evidence that the genome, the microbiome and diet all contribute to mechanisms of variation of response to simvastatin.

## Results

### Pre-treatment levels of metabolites correlated with LDL-C reduction

Two subgroups of subjects were selected from the 944 participants in the Cholesterol and Pharmacogenetics (CAP) study: 1) 100 individuals randomly selected from and representative of the entire range of LDL-C response, defined as the percentage change in LDL cholesterol from pretreatment levels (full range, FR); and 2) 24 individuals each selected from the top and bottom 10% tails of the LDL-C response distribution (total = 48) with matching of individuals from the two tails for body mass index, race, and gender (Table 1). There was a slightly lower proportion of men in the GPR than in the FR groups, but the two groups were similar in age, race, BMI, initial LDL-C, initial HDL-C, and initial total cholesterol. The two groups cannot be differentiated using these pretreatment measures.

**Table 1:** Demographics of patients included in metabolomics study.

	GPR		FR
Subjects selected from:	Good Responders	Poor Responders	Full Range Subjects
Total, n	24	24	100
Male, %	33	33	48
Body Mass Index kg/m^2^	28.5±6.4	29.1±5.1	28.5±5.1
Race % African American	25	29	30
Decrease in LDL-C, %	63.06±4.2	7.62±9.4	41.4+11.7
Age, y	60.3±13.8	53.1±12.4	53.6+12.6
Initial LDL-C, mg/dL	133.2±31.9	120.1±29.6	138.4±35.5
Initial HDL-C, mg/dL	58.17±15.46	53.66±18.57	52.6±16.5
Initial Total Cholesterol, mg/dL	219.58±39.55	201.75±32.71	215.8±39.2

Values are mean ± SD.

### A. Analysis of Full Range (FR) Samples

**Figure 1:**
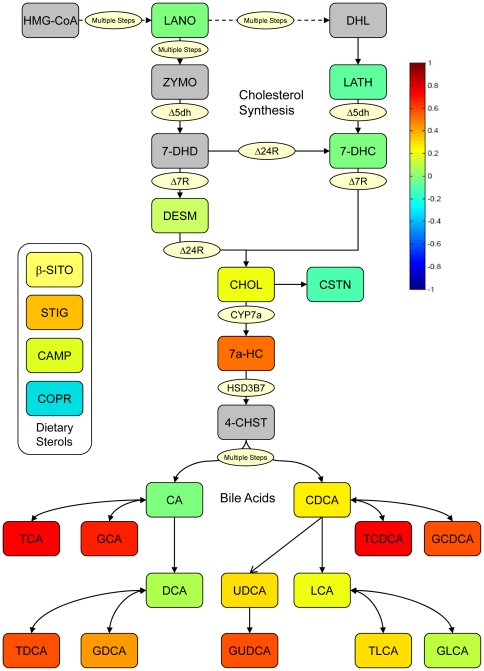
Sterol pathway map testing the association of pretreatment metabolites with change of LDL-C by statin treatment. The map was constructed using a correlation of pretreatment metabolites with change in LDL-C in FR. The color scheme corresponds to correlation strength as shown by the color bar. Red: Better response, more reduction of the metabolite. Blue: Better response, less reduction or increase of the metabolite. Correlations to change in LDL-C are given in the first row and column. The metabolites are rescaled (divided by the largest absolute value of them) to be clearer in the map. * Correlations significant by p-value, but not significant after controlling for q-value. ** Correlations significant by p-value and significant after controlling for q-value.

Using a targeted metabolomics platform with which we evaluated 12 sterols and 14 bile acids, we observed in the randomly selected (FR) subjects a very strong correlation between a lower pretreatment level of several endogenous primary and secondary bile acids—taurocholic acid (TCA), glycocholic acid (GCA), taurochenodeoxycholic acid (TCDCA), glycochenodeoxycholic acid, (GCDCA) and glycoursodeoxycholic acid (GUDCA) —and greater response to the LDL lowering effects of simvastatin (Fig. 1 and Table 2). Bile acids were the only compounds for which we observed a significant correlation with LDL reductions in this random sample from the full range of subjects.

**Table 2:** Pretreatment metabolites that correlate with change of LDL-C in FR group.

		Association with LDL-C change		
Metabolic Pathway	Metabolite	Correlation	P value	Q value
Sterol Synthesis	LANO	negative	0.8947	0.6770
	LATH	negative	0.7464	0.6212
	7.DHC	negative	0.9341	0.6770
	DESM	positive	0.6398	0.5749
	CHOL	positive	0.3984	0.4475
	CSTN	negative	0.5886	0.5696
	7.HC	positive	0.0266	0.0683
Dietary Sterols	COPR	negative	0.1692	0.3041
	B.SITO	positive	0.3330	0.4275
	STIG	positive	0.1084	0.2165
	CAMP	positive	0.4435	0.4688
Primary Bile Acids	CA	negative	0.9417	0.6770
	CDCA	positive	0.2342	0.3238
	TCA	positive	0.0023	0.0207
	GCA	positive	0.0038	0.0228
	TCDCA	positive	0.0011	0.0198
	GCDCA	positive	0.0120	0.0359
Secondary Bile Acids	DCA	positive	0.7604	0.6212
	TDCA	positive	0.0110	0.0359
	GDCA	positive	0.0607	0.1364
	LCA	positive	0.3651	0.4374
	TLCA	positive	0.2007	0.3088
	GLCA	positive	0.6022	0.5696
	UDCA	positive	0.2062	0.3088
	GUDCA	positive	0.0097	0.0359

**Figure 2:**
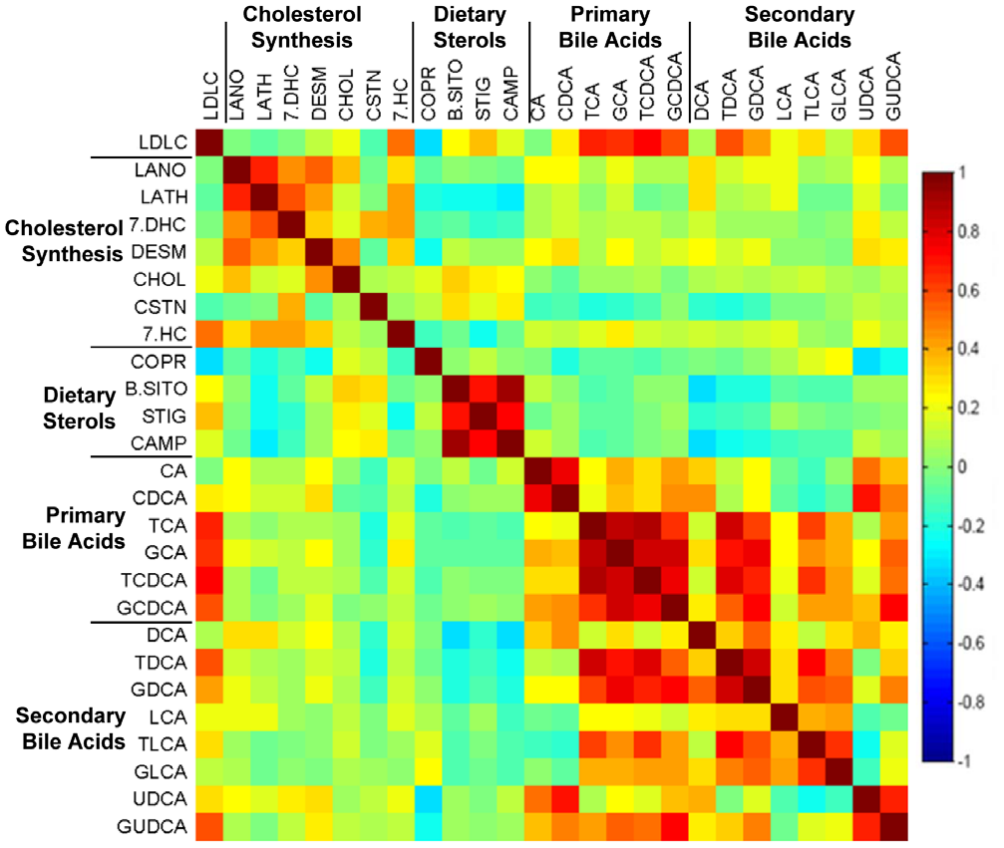
Correlation matrix for testing the association of pretreatment metabolites with a change in LDL-C by statin treatment. The correlation map shows pretreatment metabolites and change in LDL-C in FR. The color scheme corresponds to correlation strength as shown by the color bar. Red: Better response, more reduction of the metabolite. Blue: Better response, less reduction or increase of the metabolite. Correlations to changes in LDL-C are given in the first row and column. The metabolites have been rescaled (divided by the largest absolute value of them) to be clearer on the map.

A correlation matrix shows all correlations between the FR pretreatment metabolites and LDL-C response (Fig. 2). All of the metabolites within the cholesterol biosynthetic pathway except cholestanol (CSTN) were positively correlated with each other but they were not strongly correlated with LDL-C response. The dietary sterols, β-sitosterol (β-SITO), stigmasterol (STIG), and campesterol (CAMP) were highly positively correlated with each other, and were negatively correlated with most cholesterol biosynthesis metabolites, and bile acids. Coprostanol (COPR) did not correlate with the other dietary sterols and was negatively correlated with LDL-C response to statins, while β-SITO, STIG and CAMP were slightly positively correlated with LDL-C response. Most bile acids were positively correlated with each other. The primary bile acids cholic acid (CA) and chenodeoxycholic acid (CDCA) were positively correlated with most other bile acids except the secondary bile acids lithocholic acid (LCA), taurolithocholic acid (TLCA), and glycolithocholic acid (GLCA). A set of primary and secondary bile acids was positively and significantly correlated with LDL-C.

### B. Analysis of Good/Poor Responders (GPR)

**Figure 3:**
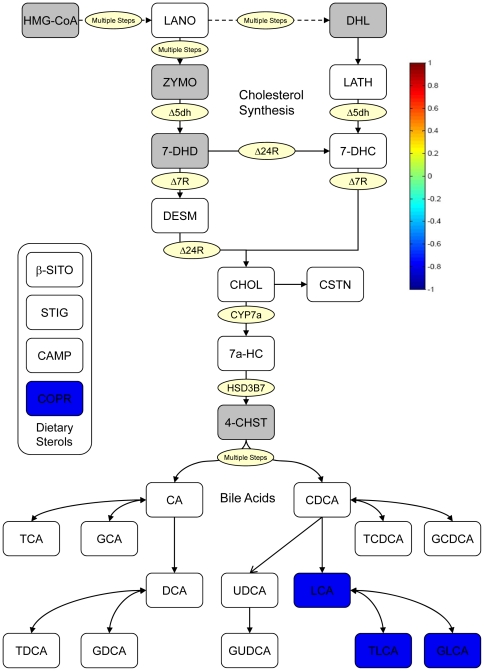
Sterol pathway map testing the association of pretreatment metabolites with changes in LDL-C from statin treatment. The map has been constructed using the correlation of pretreatment metabolites with change of LDL-C in GPR. Enzymes are represented by circles; metabolites by squares. Metabolites in grey squares were not quantified. White squares were not significantly different. The metabolites with significant p-values are colored according to the correlation relationship with blue negative and red positive. Red: Better response, more reduction of the metabolite. Blue: Better response, less reduction or increase of the metabolite. Correlations to changes in LDL-C are given in the first row and column. The metabolites have been rescaled (divided by the largest absolute value of them) to be clearer on the map. * Correlations significant by p-value, but not significant after controlling for q-value. ** Correlations significant by p-value and significant after controlling for q-value.

We next evaluated samples from the tails of the spectrum of LDL-C response, that is, subsets of the best and worst responders to simvastatin therapy (Fig. 3). As shown in Figure 3 and Table 3, we observed a strong relationship between the level of reduction in LDL-C levels and higher pretreatment levels of the secondary bile acids, LCA, TLCA and GLCA, as well as COPR which is produced in the intestine by enteric bacterial reduction of endogenous cholesterol.

**Table 3:** Pretreatment metabolites correlated with treatment outcomes in patients selected from the ends (comparing good and poor responders).

Metabolic Pathway	Metabolite	Correlation	P value	Q value
Sterol Synthesis	LANO	positive	0.9037	0.9596
	LATH	negative	0.4350	0.7932
	7.DHC	negative	0.3388	0.7932
	DESM	negative	0.1498	0.4680
	CHOL	negative	0.1170	0.4179
	CSTN	positive	0.8517	0.9596
	7.HC	negative	0.8345	0.9596
Dietary Sterols	B.SITO	negative	0.9212	0.9596
	CAMP	positive	0.7004	0.9596
	COPR	negative	0.0242	0.2016
	STIG	positive	0.3851	0.7932
Primary Bile Acids	CA	positive	0.4126	0.7932
	CDCA	positive	0.0991	0.4129
	TCA	negative	0.4628	0.7932
	GCA	negative	0.8398	0.9596
	TCDCA	negative	0.7576	0.9596
	GCDCA	negative	0.7739	0.9596
Secondary Bile Acids	DCA	negative	0.6938	0.9596
	UDCA	negative	0.9915	0.9915
	LCA	negative	0.0443	0.2770
	TDCA	negative	0.0568	0.2841
	GDCA	negative	0.2376	0.6599
	GUDCA	negative	0.4759	0.7932
	TLCA	negative	0.0198	0.2016
	GLCA	negative	0.0234	0.2016

**Figure 4:**
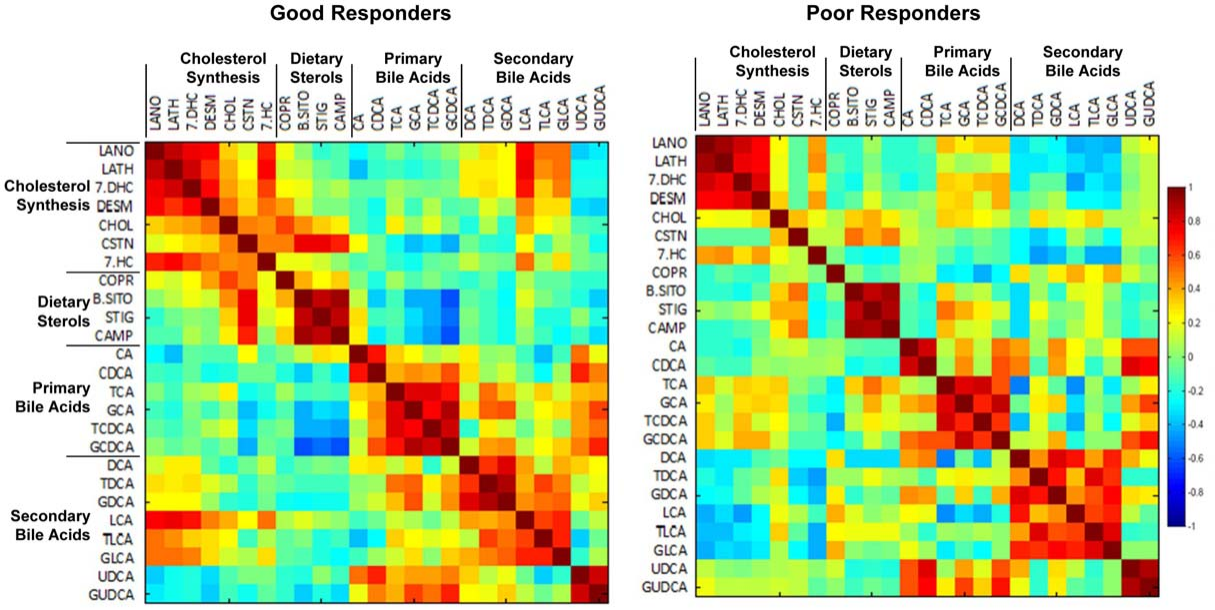
Correlation matrices of pretreatment sterol metabolites in good and poor responders. The differences between the two matrices reflects the differences between the groups responses to statin treatment. The color scheme corresponds to correlation strength as shown by the color bar. Red: Better response, more reduction of the metabolite. Blue: Better response, less reduction or increase of the metabolite. The metabolites have been rescaled (divided by the largest absolute value of them) to be clearer on the map.

Separate correlation matrices for the good and the poor responders show all correlations of pretreatment metabolites within each group (Fig. 4). There were some strong positive correlations within the cholesterol biosynthesis metabolites, dietary sterols, and bile acids in both good and poor responders. COPR had a positive correlation with the dietary sterols in the good responders and no correlation in the poor responder subjects. Bile acids were also different between the good and poor responders, with primary bile acids (TCA, GCA, TCDCA, GCDCA) changing from no correlation with cholesterol biosynthesis and negative correlation with dietary sterol metabolites in good responders to a slightly positive correlation in poor responders. Conversely, secondary bile acids (deoxycholic acid DCA, taurodeoxycholic acid TDCA, GDCA, LCA, TLCA, and GLCA) showed a positive correlation with cholesterol biosynthesis metabolites in good responders and a negative correlation in the poor responders.

### Pre-treatment levels of metabolites correlated with on-treatment simvastatin concentration

Pretreatment concentrations of two bile acids, CDCA and DCA, were positively correlated with on-treatment plasma simvastatin acid concentrations in the FR subjects (Table 4). Neither pre- nor post-treatment level of LDL-C was significantly correlated with plasma simvastatin concentration in this study (Table 4 and data not shown) or in the entire CAP study (data not shown).

**Table 4:** Pretreatment sterol metabolites and bile acids correlated with simvastatin concentrations.

	Metabolite	Correlation	P value	Q value
Full Range	CDCA	0.21	0.039	0.09
	DCA	0.24	0.019	0.09
	LDL-C	0.038	0.71	0.21
Good Responders	CDCA	−0.47	0.035	0.2
	LCA	0.47	0.033	0.2
Poor Responders	LANO	0.44	0.034	0.2
	TCA	0.49	0.017	0.17
	TCDCA	0.42	0.043	0.21
	DCA	−0.54	0.0078	0.17
	LCA	−0.5	0.014	0.17

Several bile acids were correlated with simvastatin acid concentrations in the good and the poor responders, with borderline significant q-values (Table 4). These include negative correlations to CDCA and positive correlations to LCA in good responders; and, in poor responders, negative correlations to both secondary bile acids (DCA and LCA) that were positively correlated to simvastatin acid levels in good and normal range responders.

### Pre-treatment levels of several metabolites correlated with SLCO1B1 gene polymorphisms

We tested the FR subjects for associations of plasma lipid metabolites with SNP rs4149056 in the gene encoding the organic anion transporter SLCO1B1. In the full CAP study population, this SNP was strongly associated with fasting simvastatin acid concentration (p<0.0001, r = 0.23, R. Krauss, personal communication), consistent with earlier observations [16]. We were interested in determining whether SNP rs4149056 was associated with levels of sterol/bile acid metabolites whose pre-treatment concentrations had been correlated with simvastatin LDL response. Our analysis (Table 5) revealed that seven bile acids showed nominally significant associations with this SNP, such that the minor allele was associated with higher plasma levels. These include two secondary bile acids, LCA and TLCA, which we identified as markers for greater response to simvastatin lowering of LDL-C levels in good responders. LDL-C reduction in FR subjects was also correlated with post simvastatin treatment levels of LCA and TLCA (data not shown). The minor allele of rs4149056 was also associated with increased plasma levels of 7α-hydroxycholesterol, the product of the rate-limiting step in synthesis of bile acids from cholesterol (Table 5).

**Table 5:** Association of SLCO1B1 SNP rs4149056 with pretreatment measurements for sterols and bile acids in CAP participants.

Metabolites	Estimate	P value	Q value
LANO	0.15	0.13	0.18
LATH	0.11	0.29	0.28
7.DHC	0.13	0.2	0.24
DESM	0.071	0.48	0.37
CHOL	0.15	0.14	0.18
CSTN	0.017	0.87	0.53
**7.HC**	**0.21**	**0.039**	**0.088**
COPR	0.085	0.4	0.34
B.SITO	0.0038	0.97	0.55
STIG	−0.12	0.23	0.25
CAMP	−0.057	0.57	0.37
**CA**	**0.22**	**0.026**	**0.088**
CDCA	0.11	0.26	0.27
**TCA**	**0.22**	**0.03**	**0.088**
**GCA**	**0.26**	**0.0084**	**0.088**
**TCDCA**	**0.23**	**0.023**	**0.088**
GCDCA	0.16	0.12	0.18
DCA	0.064	0.53	0.37
TDCA	0.16	0.12	0.18
GDCA	0.17	0.088	0.17
**LCA**	**0.21**	**0.04**	**0.088**
**TLCA**	**0.22**	**0.027**	**0.088**
GLCA	0.065	0.52	0.37
UDCA	0.056	0.58	0.37
GUDCA	0.087	0.39	0.34

We also analyzed a second SLCO1B1 SNP (rs2306283, an N130D coding variant) that was not associated with plasma simvastatin acid levels and found a significant correlation only with plasma stigmasterol concentration (Table 6).

**Table 6:** Association of SLCO1B1 SNP rs2306283 with pretreatment measurements of sterols and bile acids in CAP participants.

Metabolites	Estimate	P value	Q value
LANO	−0.11	0.29	0.6
LATH	−0.02	0.84	0.6
7.DHC	−0.027	0.79	0.6
DESM	0.046	0.65	0.6
CHOL	−0.15	0.15	0.6
CSTN	−0.049	0.63	0.6
7.HC	−0.08	0.43	0.6
COPR	−0.076	0.46	0.6
**B.SITO**	**−0.17**	**0.09**	**0.58**
**STIG**	**−0.28**	**0.0045**	**0.086**
**CAMP**	**−0.19**	**0.064**	**0.58**
CA	−0.038	0.71	0.6
CDCA	−0.058	0.57	0.6
TCA	−0.042	0.68	0.6
GCA	−0.077	0.45	0.6
TCDCA	−0.043	0.68	0.6
GCDCA	−0.039	0.7	0.6
DCA	−0.031	0.76	0.6
TDCA	0.018	0.86	0.6
GDCA	−0.017	0.87	0.6
LCA	−0.056	0.59	0.6
TLCA	0.015	0.89	0.6
GLCA	−0.027	0.79	0.6
UDCA	0.08	0.43	0.6
GUDCA	0.019	0.85	0.6

## Discussion

Statins lower plasma LDL-C by blocking the activity of HMG-CoA reductase, thereby decreasing the synthesis of cholesterol and modifying downstream metabolic pathways. In our previous study we evaluated over 300 lipid species within eight lipid classes and found that baseline cholesterol ester and phospholipid metabolites correlated with LDL-C response to treatment [17]. This study builds on our previous work to investigate sterol metabolism in more depth and beyond effects on HMG-CoA reductase. We used a selective analytic platform to assay levels of sterol metabolites in plasma of participants in a trial of simvastatin treatment in order to identify biomarkers that correlate with statin lipid-modifying efficacy. We investigated metabolite levels from both the best and worst responders as well as from subjects with a full range of response. Doing so allowed us to explore and verify the effects of statin treatment in two population subsets representing a wide range of LDL-C responses. The FR subset provided an assessment of the predictors of statin response that is applicable to a wide range of statin-treated patients. On the other hand, the GPR subset provided a means to explore the differences in metabolite predictors of the highest and lowest responses to drug therapy, although the results may be applicable to only a small subset of patients who take simvastatin. We identified a positive correlation between LDL-C response and specific primary and secondary bile metabolites including TCA, GCA, TCDCA, GCDCA and GUDCA. In addition, there was a negative correlation in the GPR group with secondary bile acids produced by intestinal bacteria. These enteric bacterially produced bile acids included lithocholic acid (LCA) and the conjugated derivatives glycolithocholic acid (GLCA) and taurolithocholic acid (TLCA). The observation that different secondary bile acids were inversely associated with LDL-C response in the two study subsets may signify differing specific relationships among extreme responders compared with the remainder of the population.

In this study there were no strong correlations between pretreatment bile acid metabolites and HDL-C response to simvastatin (data not shown), which further supports the specificity of the relationship between the concentration of the bile acid metabolites and LDL-C response.

Bile acids in plasma act as detergents to solubilize hydrophobic nutrient molecules including cholesterol, and other endogenous and exogenous sterols. Accordingly, the complex interplay of endogenous primary and bacterial metabolized secondary bile acids can affect the retention and metabolism of plasma cholesterol. Bile acids are known to be important endocrine signals, functioning in the systemic control of lipid levels, muscle function and immune cell regulation [18]. It has not escaped our attention that all of these pathways are affected by statins, either as therapeutic target or side effects, suggesting that bile acids may be important mediators of statin activities.

**Figure 5:**
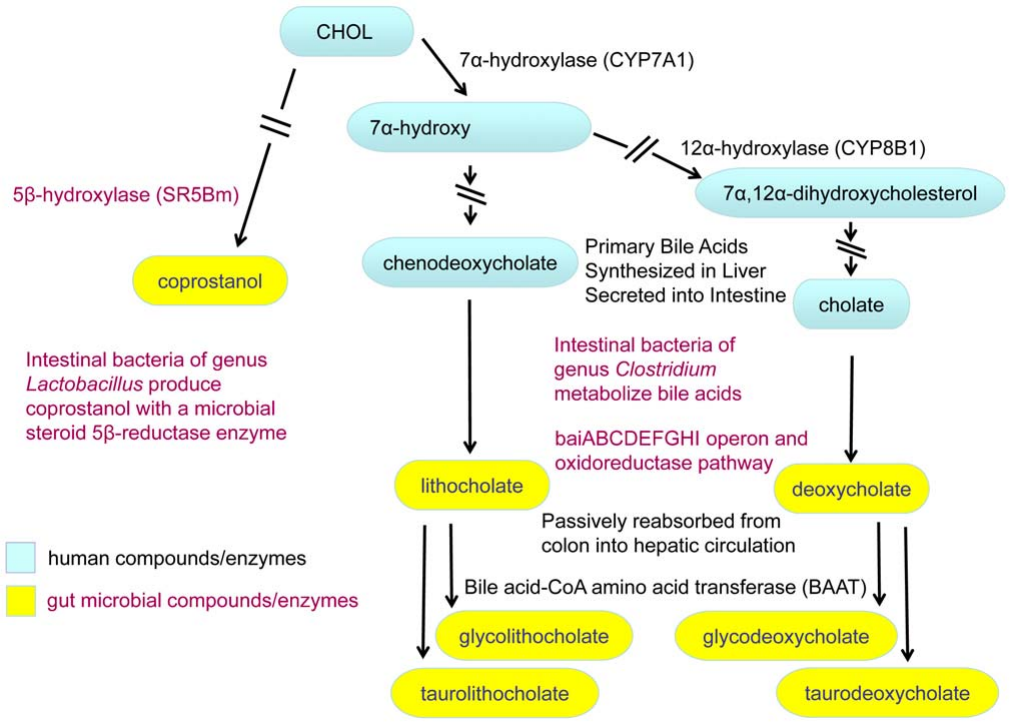
Active cholesterol metabolites are produced by interspecies biosynthetic pathways. Bile acids are the main metabolites of cholesterol (CHOL). Primary bile acids (blue ovals; cholic acid (CA) and chenodeoxycholic acid (CDCA)) are produced by endogenous enzymes in the liver and are modified by bacteria of the genus *Clostridia* colonizing the gut to form secondary bile acids (yellow ovals; lithocholic acid (LCA) and deoxycholic acid [18]). Arrows broken by double lines represent multiple enzymatic steps and only the genes encoding the rate limiting enzymes are listed. The bacterial operon baiABCDEFGHI encodes eight enzymes and a bile acid transporter that together form the pathway for synthesis of secondary bile acids. The amino acids glycine and taurine are conjugated to primary and secondary bile acids in the liver by the host encoded bile acid-CoA amino acid transferase (BAAT) to form tauro- and glycolithocholic acid (TLCA, GLCA) and tauro- and glycodeoxycholic acid (TDCA GDCA). Gut bacteria of genus *Lactobacillus* catalyze the conversion of cholesterol metabolites to coprostanol (COPR) and can limit the intestinal absorption of cholesterol.

In addition to secondary bile acids, we observed a correlation between response to simvastatin and higher pre-treatment levels of coprostanol (COPR) (Figs. 1, 2, and 3). This compound is used as an environmental marker for the presence of human fecal matter and is produced by intestinal bacteria by hydroxylation of cholesterol (Fig. 5). The cholesterol to coprostanol ratio has been used as a measure of ability to remove cholesterol from circulation, and has been shown to be controlled by the amount of coprostanoligenic bacteria in the gut [19]. Specific strains of *Lactobacill*ae bacteria isolated from fermented dairy products have been characterized by their ability to convert cholesterol to coprostanol and have been suggested as probiotic alternatives for reducing cholesterol levels [20]. Our data suggest that patients with higher pretreatment levels of coprostanoligenic bacteria will respond more robustly to the LDL-C lowering effects of simvastatin. Other investigators have linked enteric bacteria metabolism with cardiovascular changes and disease outcome in animal models using metabolomics [15]. The work presented here expands that concept and provides a link between enteric bacterial metabolism and therapeutic outcome of simvastatin treatment in humans.

Another potential basis for the relationship between gut microbial metabolism and statin efficacy is an effect on drug absorption or other factors affecting drug pharmacokinetics [12] or drug pharmacodynamics.

Our analyses identified primary and secondary bile acids for which pretreatment concentrations were correlated with on-treatment plasma simvastatin acid levels. Primary bile acids (CA and CDCA) and simvastatin are all metabolized by the P450 enzyme CYP3A4 [21], [22]. Simvastatin is transported by multidrug resistance gene 1 (MDR1, ABCB1) P-glycoprotein, multidrug resistance-associated protein 2 (MRP2, ABCC2), and organic anion-transporting polypeptide 1B1 [23], [24]. Polymorphisms in these proteins are known to influence simvastatin pharmacokinetics [25], [26], [27]. These transporters are also responsible for the transport of bile acids in the gastrointestinal tract and liver [28], [29]. At the same time simvastatin and bile acids also regulate the expression of these transporters [28], [30], [31], [32]. Interactions between simvastatin and selected bile acids competing for membrane transport may be responsible for some of the off target effects of the drug [33].

We speculate that the correlations between plasma simvastatin and bile acids occurred because transport of these compounds to or from the plasma is limiting. Therefore genetic polymorphisms which alter the activity or amount of hepatic transporters could result in changes in both simvastatin and bile acid concentrations. Genetic analyses of the CAP population, including the subjects in this study, have identified correlations between a *SLCO1B1* SNP and plasma levels of simvastatin acid as well as specific primary and secondary bile acids. The SLCO1B1 transporter is of particular interest because it is known to transport simvastatin from plasma into the liver, and SNP rs4149056 has been associated with statin-induced myopathy in individuals treated with high dose simvastatin [34]. The present results are generally consistent with those published recently from a much smaller study population [16] and demonstrate that SLCO1B1 plays an important role in hepatic uptake of bile acids as well as simvastatin. Further, they raise the possibility that competition between simvastatin and bile acids for this transporter may influence both the pharmacokinetics and pharmacodynamics of simvastatin, and possibly risk of muscle toxicity. It should be pointed out however that the findings from the present study may not be applicable to other statins, whose interactions with bile acid metabolism and transport may differ from those of simvastatin.

The present findings point to the utility of metabolomic surveys for identifying predictors of clinical response that may have implications for assessing efficacy of this widely-used class of drugs. We have observed that the pretreatment levels of bile acids derived from gut bacteria and nutrient inputs are correlated with response to simvastatin. It is becoming increasingly clear that gut microbial symbiots are critical for normal digestion and defense, and also play an important role in development of disease [15] and in metabolizing orally ingested therapeutics [12]. There is increasing recognition that intestinal bacteria can metabolize drugs and alter an individual's response to drug treatment depending on specific bacterial strains present [35]. In an interesting corollary to our work, Ridlon et al. have suggested that probiotics, by altering intestinal microflora, can alter the enterohepatic circulation of secondary bile acids [36]. In addition, Wang et al. recently have implicated enteric bacteria and phosphatidylcholine metabolism in the pathogenesis of cardiovascular disease [15]. We suggest that our findings warrant further evaluation of interactions of specific markers for gut microbiota and therapeutic response to statins. Identification of the basis for such interactions may in turn lead to dietary or other interventions that can improve statin efficacy by altering gut microflora.

## Methods

### Subjects

Plasma samples were analyzed from participants in the Cholesterol and Pharmacogenetics (CAP) study—a trial in which 944 Caucasian and African-American men and women with total cholesterol levels of 160–400 mg/dL were treated with simvastatin 40 mg/d for 6 weeks. This study was designed to examine genetic and non-genetic factors affecting the response to simvastatin therapy in healthy, drug-naïve patients [37]. Participants were followed for a total of 6 weeks on simvastatin therapy (40 mg at bedtime) and were seen at clinic visits conducted at 2-week intervals. Blood specimens from each subject were obtained after overnight fast at the screening visit, after a 2-week placebo run-in (enrollment visit), and following 4 and 6 weeks of simvastatin administration. Samples used in this study were collected pretreatment and at 6 weeks of therapy. Simvastatin concentrations were analyzed in the samples collected at 6 weeks. Compliance was assessed by pill count every 2 weeks and averaged more than 95%. Overall, treatment with simvastatin lowered LDL-C by 54 mg/dl and increased HDL cholesterol (HDL-C) by 2 mg/dl. The magnitude of the lipid and lipoprotein responses, however, differed among participants according to a number of phenotypic and demographic characteristics [37]. Data on dietary intake was not collected, but subjects were instructed not to change their diet. IRB approval was granted by the participating institutions and informed consent was obtained from all participants in CAP.

Two subgroups of subjects were selected for study. The first (good/poor responders, GPR) consisted of 24 individuals selected from the top 10% of the LDL-C response distribution who were matched for body mass index (BMI), race, and gender to 24 individuals in the lowest 10% of responders, with response to therapy defined as the percentage change in LDL cholesterol from pretreatment levels. A second set of 100 individuals (full range, FR) was randomly selected from the entire CAP study, excluding subjects who had been selected for the initial GPR group. These subjects are representative of the population for age, race, gender, and BMI. Metabolomic analyses of statin-induced changes in the fatty acid content of the major lipid classes in the FR group have been reported recently [17].

As shown in Table 1, good responders were slightly but not significantly older than poor responders, with marginally higher initial cholesterol, HDL-C, apolipoprotein AI (apoAI), and apolipoprotein B (apoB) levels The FR group, with the broad response range, had a slightly higher percentage of men than Group 1, but was matched for race, age, and initial cholesterol levels.

### Laboratory measurements

Plasma LDL-C, HDL-C, apoAI and apoB were measured as described previously (Simon et al., 2006). Lipid extraction, solid-phase extraction, and capillary gas-liquid chromatography were used for quantitative analysis of sterols according to the method of Phillips et al. [38]. The analysis was conducted using an Agilent 5975 GC/MSD. Bile acids were extracted using a sample preparation involving protein precipitation as described in Tagliacozzi et al. [39]. Analysis separations were performed by the Agilent 1200 RRLC (rapid resolution liquid chromatograph) using a Zorbax 1.8 micron column; bile acids were detected with the 4000 Qtrap (Applied Biosystems) by monitoring the analytes under multiple reaction-monitoring mode as described in Burkard et al. [40]. The following sterols and bile acids were detected and quantified: campesterol (CAMP), chenodeoxycholic acid (CDCA), cholestanol (CSTN), 4-cholesten-7a-ol-3-one (4-CHST), cholesterol, cholic acid (CA), coprostanol (COPR), 7-dehydrocholesterol (7-DCH), deoxycholic acid (DCA), desmosterol (DESM), glycochenodeoxycholic acid (GCDCA), glycocholic acid (GCA), glycodeoxycholic acid (GDCA), glycolithocholic acid (GLCA), glycoursodeoxycholic acid (GUDCA), 7α-hydroxycholesterol (7α-HC), lanosterol (LANO), lathosterol (LATH), lithocholic acid (LCA), β-sitosterol (β-SITO), stigmasterol (STIG), taurochenodeoxycholic acid (TCDCA), taurocholic acid (TCA), taurodeoxycholic acid (TDCA), taurolithocholic acid (TLCA), and ursodeoxycholic acid (UDCA).

The levels of activated metabolite of simvastatin, simvastatin acid, were determined by mass spectrometry as described elsewhere [41]. Analysis of the *SLCO1B1* rs4149056 single nucleotide polymorphism (SNP) was performed by BeadArray technology (Illumina, San Diego, CA).

### Statistical analyses

We tested the correlation of pretreatment metabolites with drug response. Metabolites were log-transformed before correlation testing. For FR subjects, Spearman's correlation coefficient was used to obtain correlation coefficients between the pretreatment level of each metabolite with changes in LDL-C or HDL-C. Because changes in LDL-C and HDL-C are correlated with pretreatment LDL-C and HDL-C concentrations, respectively, the changes of these measurements were adjusted for their pretreatment levels \. Correlations among pretreatment metabolites were obtained by deriving Spearman's correlation coefficient between each pair. For GPR subjects, Wilcoxon rank sum tests were used to test differences in pretreatment metabolites between good responders and poor responders. Correlation maps were constructed using the algorithm in Ayroles et al. [42]. The degree of correlation between each two metabolites was color-coded. Metabolites were listed in the order of the pathway maps for sterol metabolism.
