# Correction: Enteric Microbiome Metabolites Correlate with Response to Simvastatin Treatment

**DOI:** 10.1371/annotation/8e8e95ca-1ac3-4acf-abcb-223cd11ac1c1

**Published:** 2013-05-06

**Authors:** Rima Kaddurah-Daouk, Rebecca A. Baillie, Hongjie Zhu, Zhao-Bang Zeng, Michelle M. Wiest, Uyen Thao Nguyen, Katie Wojnoonski, Steven M. Watkins, Miles Trupp, Ronald M. Krauss

There were errors in Figures 1 and 3, and in the legends of Figures 1,2,3, and 4.

The correct versions of the Figures can be found here:

Figure 1: 

**Figure:**
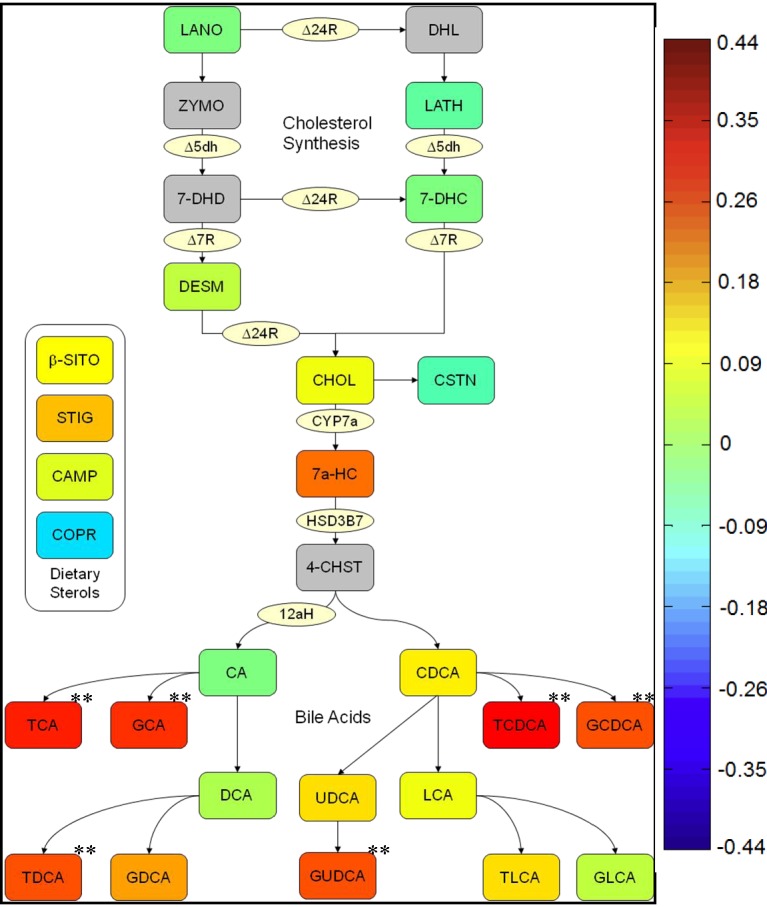


Figure 3: 

**Figure:**
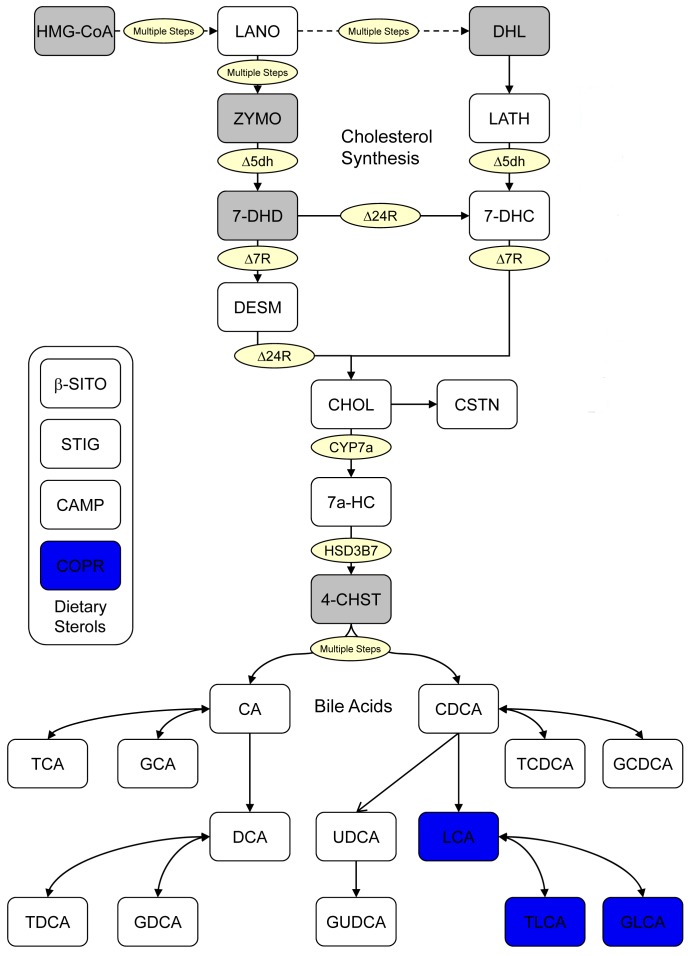


The correct versions of the legends can be found here:

**Figure 1. Sterol pathway map testing the association of pretreatment metabolites with change of LDL-C by statin treatment.** The map was constructed using a correlation of pretreatment metabolites with change in LDL-C in FR. The color scheme corresponds to correlation strength as shown by the color bar. Red: Lower level at baseline, greater response. Blue: Higher level at baseline, greater response. Enzymes are represented by circles; metabolites by squares. Metabolites in grey squares were not quantified. White squares were not significantly different. ** Correlations significant after controlling for q-value.

**Figure 2. Correlation matrix for testing the association of pretreatment metabolites with a change in LDL-C by statin treatment.** The correlation map shows pretreatment metabolites and change in LDL-C in FR. The color scheme corresponds to correlation strength as shown by the color bar. Red: Lower level at baseline, greater response. Blue: Higher level at baseline, greater response. Correlations to changes in

LDL-C are given in the first row and column. These correlations have been rescaled (divided by the largest absolute value of them) to be clearer on the map.

**Figure 3. Sterol pathway map testing the difference in pretreatment metabolites between good and poor responders in GPR.** Enzymes are represented by circles; metabolites by squares. Metabolites in grey squares were not quantified. White squares were not significantly different. The metabolites with significant p-values are colored blue indicating higher baseline level of these metabolites are associated with greater response.

**Figure 4. Correlation matrices of pretreatment sterol metabolites in good and poor responders.** The differences between the two matrices reflects the differences between the groups responses to statin treatment. The color scheme corresponds to correlation strength as shown by the color bar. 

